# Hearing Loss and Risk of Overall, Injury-Related, and Cardiovascular Mortality: The Kangbuk Samsung Health Study

**DOI:** 10.3390/jcm9051415

**Published:** 2020-05-10

**Authors:** Woncheol Lee, Yoosoo Chang, Hocheol Shin, Seungho Ryu

**Affiliations:** 1 Department of Occupational and Environmental Medicine, Kangbuk Samsung Hospital, Sungkyunkwan University School of Medicine, Seoul 03181, Korea; doctor.oem@gmail.com; 2 Center for Cohort Studies, Total Healthcare Center, Kangbuk Samsung Hospital, Sungkyunkwan University School of Medicine, Seoul 04514, Korea; hcfm.shin@samsung.com; 3 Department of Clinical Research Design & Evaluation, SAIHST, Sungkyunkwan University, Seoul 06351, Korea; 4 Department of Family Medicine, Kangbuk Samsung Hospital, Sungkyunkwan University School of Medicine, Seoul 03181, Korea

**Keywords:** overall mortality, cardiovascular mortality, cohort study, injury-related mortality, hearing loss

## Abstract

Hearing loss (HL) has been related to cardiovascular risk factors as well as prevalence of cardiovascular disease itself. We evaluated the association of HL with overall, injury-related, and cardiovascular mortality. A cohort study included 580,798 Korean adults (mean age: 39.7) who attended a screening exam between 2002 and 2016 with a follow-up of up to 17 years. HL was defined as a pure-tone average of thresholds at 0.5, 1.0, and 2.0 kHz ≥25 dB (decibels) in the better ear and further categorized into mild (25–<40 dB) and moderate-to-severe (≥40 dB). Overall and cause-specific mortality was ascertained through linkage to national death records. During median follow-up of 8.4 years, 6581 overall deaths, 977 cardiovascular deaths, and 1161 injury-related deaths were identified. Compared to participants with normal hearing, multivariable-adjusted hazard ratios (HRs) with 95% confidence intervals (95% CIs) for overall mortality among participants with mild and moderate-to-severe HL were 1.13 (1.05–1.21) and 1.30 (1.16–1.46), respectively. Corresponding HRs (95% CIs) for cardiovascular mortality were 1.32 (1.10–1.58) and 1.53 (1.16–2.01), respectively, and corresponding HRs (95% CIs) for injury-related mortality were 1.03 (0.81–1.31) and 1.64 (1.13–2.36), respectively. In this large cohort, HL was positively and independently associated with overall, cardiovascular, and injury-related mortality. A significantly elevated risk of cardiovascular mortality started from mild HL.

## 1. Introduction

Hearing loss (HL) is one of the most prevalent chronic diseases and is a leading cause of disease burden globally [1]. In 2015, approximately 466 million people, over 5% of the world’s population, were estimated to have disabling HL [2].

Multiple studies have suggested that HL adversely affects physical and social function, reduces hearing-related quality of life, and increases the risk of depressive disorders, cognitive decline, and fractures or injuries [3,4,5,6]. Previous epidemiological studies have also investigated whether HL increases mortality, but with inconsistent results. Some studies demonstrated the positive association between HL and mortality, whereas others reported no association [7,8,9,10,11,12,13,14,15]. Many studies are limited by a small number of subjects and by defining HL through self-report instead of audiometric tests. This may have introduced measurement errors or misclassification and reduced the degree of association. Furthermore, most studies are mainly conducted in elderly patients and focus on overall mortality without further detailed information on cause-specific mortality. HL appears to relate to individual and multiple cardiovascular risk factors, including smoking, diabetes, hypertension, hyperlipidemia, and obesity, and it is often associated with cardiovascular disease itself [16,17,18,19,20,21,22]. However, it is still unclear whether HL predicts future cardiovascular events or cardiovascular mortality. This is important in order to determine whether preventive measures and treatment of cardiovascular disease are needed in patients with HL.

Therefore, we examined whether HL is associated with overall, cardiovascular, and injury-related mortality in a large-scale cohort study of 580,798 Korean adults who attended a health check-up program. The study accounts for change in HL, cardiovascular risk factors, and other covariates during follow-up.

Key points: Question: Is there an association of hearing loss (HL) with overall, injury-related, and cardiovascular mortality among apparently healthy, relatively young people? Findings: In this cohort study that included 580,798 Korean adults (mean age: 39.7), a significantly increased risk of all-cause and cardiovascular mortality was observed in participants with HL, with graded association starting from mild hearing loss, and the risk of injury-related mortality was significantly elevated with moderate-to-severe HL. Meaning: HL was positively associated with overall, injury-related, and cardiovascular mortality, with an increased risk of cardiovascular mortality observed in individuals with even mild HL. Further research is needed to determine whether proper screening and management for cardiovascular diseases in patients with HL are needed to reduce cardiovascular mortality.

## 2. Methods

### 2.1. Study Population 

The present cohort study was performed using a subsample of the Kangbuk Samsung Health Study, a cohort study of Korean men and women who attended an annual or biennial health check-up program at one of the Kangbuk Samsung Hospital Total Healthcare Centers in Seoul and Suwon, South Korea, as described previously [23]. The present study included participants who underwent a screening exam between 2002 and 2016 (*N* = 581,526). 

**Figure 1:**
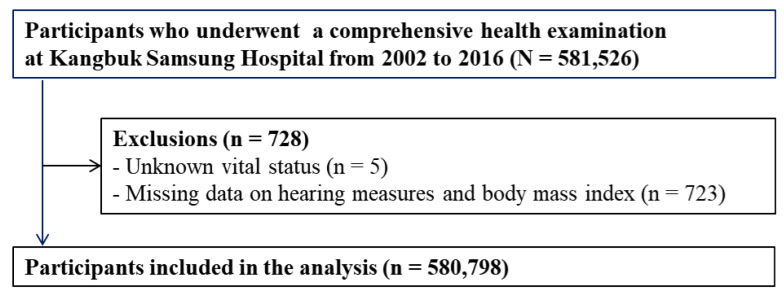
Flowchart of study participants.

A total of 728 subjects were excluded due to the following criteria at baseline (Figure 1): unknown vital status (*N* = 5), and missing information on hearing measures and anthropometry (*N* = 723). A total of 580,798 participants were included in the final analysis. 

The Institutional Review Board of Kangbuk Samsung Hospital approved this study (IRB No. KBSMC 2020-01-023), and waived the requirement for informed consent due to the use of anonymized retrospective data that were collected as a routine part of the health checkup program and already linked to mortality data from the Korea National Statistical Office (KNSO) [24,25,26].

### 2.2. Data Collection

Hearing tests and other examinations, at baseline and follow-up visits, were conducted at Kangbuk Samsung Hospital Total Healthcare Centers. Data regarding demographic characteristics, lifestyle factors, and medical history were collected using a standardized, self-administered questionnaire. Participants were categorized as never, ex-, and current smokers. Average alcohol intake per day was categorized as either less than 20 grams or 20 grams or more. The weekly frequency of moderate or vigorous physical activity (from April 2009 to 2018) [27,28] and the weekly frequency of physical activity that lasted long enough to generate perspiration (from 2002 to March 2009) [27] were assessed via self-administered questionnaire and were categorized into none, <3 times per week, and ≥3 times per week [27,29].

Anthropometry and sitting blood pressure (BP) were measured by trained nurses. Obesity was defined as body mass index (BMI) ≥25 kg/m^2^, Asian-specific criteria for diagnosis of obesity [30]. Hypertension was defined as BP ≥140/90 mmHg, or the use of BP-lowering medication.

### 2.3. Laboratory Measurements

Fasting blood tests covered lipid profiles, glucose, insulin, and high-sensitivity C-reactive protein (hsCRP), as previously described [23]. The homeostatic model assessment–insulin resistance (HOMA-IR) was calculated as follows: fasting blood insulin (uU/mL) × fasting blood glucose (mg/dl)/450. Diabetes mellitus was defined as fasting serum glucose level ≥126 mg/dl or current use of anti-diabetic medication.

### 2.4. Audiometric Measurements 

At baseline and follow-up visits, pure-tone audiometric testing was performed by trained audiometric technicians using a GSI 67 audiometer (Grason-Stadler (GSI), Bedford, MA, USA) equipped with TDH-39 supra-aural earphones (Telephonics Co., Farmingdale, New York, USA) in a dedicated sound-attenuating booth [17]. Pure-tone air conduction thresholds were measured in decibels (dB) hearing level (HL) for both ears at 0.5, 1.0, and 2.0 kHz. HL was defined as a pure-tone average of thresholds at 0.5, 1.0, and 2.0 kHz ≥ 25 dB in the better ear and were further categorized as mild (25 to <40 dB) and moderate-to-severe (≥40 dB).

The Korean Occupational Safety and Health Act 28 requires an annual hearing test at 3.0 and 4.0 kHz in addition to the regular frequencies (0.5, 1.0, and 2.0 kHz) for employees who are exposed to equivalent sound pressure level of 85 dB(A) in the workplace over an 8 h work day. Hearing tests at 3.0 and 4.0 kHz, in addition to routine testing at 0.5, 1.0, and 2.0 kHz, were measured in only a small proportion of participants who met the above criteria. Thus, these tests were not used to define HL, but were used as a proxy marker of occupational noise exposure.

### 2.5. Mortality Ascertainment

Vital status until December 2018 was based on national death certificate data from the Korea National Statistical Office (KNSO) [24,25]. In Korea, all deaths of Koreans are reported to the KNSO. Cause of death was determined on the basis of the cause listed on each death certificate, which was reported according to the International Classification of Diseases and Related Health Problems 10th Revision (ICD-10). Concordance between the cause of death on the death certificate and patient diagnosis in the medical utilization data was 72.2% for overall deaths [31], Cardiovascular mortality was defined as ICD-10 codes I00 to I99, and injury-related mortality was defined as death from unintentional injury (ICD-10 codes S00 to T98) [32].

### 2.6. Statistical Analysis

The baseline characteristics are shown according to HL categories using descriptive summary statistics including mean (standard deviation: SD), median (interquartile range), or number (percentage), as appropriate. 

Each study subject was followed from baseline exam until either death or the end of 2018, whichever came first. For analysis of cause-specific mortality, individuals who died of other causes were censored at the date of death. Cox-proportional hazards regression analyses, using age as the time-scale, were performed to estimate hazard ratios (HRs) and 95% confidence intervals (CIs) for overall and cause-specific mortality. Age at which subjects underwent their first health checkup exam (left truncation) and the age at which subjects exited the analysis at the date of death or on 31 December 2018 were used in analyses. The proportional hazards assumption was evaluated by examining graphs of estimated log (-log (SURVIVAL)), and no major violations were found.

Initially, models were adjusted for age (as time scale) and sex, and then further adjusted for additional variables that might confound the relationships among hearing categories and mortality: study center (Seoul, Suwon), year of exam (1-year categories), smoking status (never, ex-, current, or unknown), regular exercise (<3, ≥3 times a week, or unknown), alcohol intake (<20, ≥20 grams per day, or unknown), BMI (continuous), education level (<community college graduate, ≥community college graduate, or unknown), history of diabetes, history of hypertension, history of cancer, history of cardiovascular disease, and medication for dyslipidemia (multivariable-adjusted model). Time-dependent analyses were conducted in which changes in hearing status and other confounders during follow-up were updated as time-varying covariates in the models. To further explore the shape of the dose–response relationship of hearing level with mortality, restricted cubic splines with knots were used at the 5th, 27.5th, 50th, 72.5th, and 95th percentiles of hearing level distribution.

Sensitivity analysis was performed using HL defined as a pure-tone average of thresholds at 0.5, 1.0, and 2.0 kHz ≥25 dB in either the left or right ear instead of the better ear. Additionally, subgroup analyses were performed by exposure to occupational noise (no vs. yes). Interactions between hearing categories and subgroup characteristics were tested using likelihood ratio tests that compared models with and without multiplicative interaction terms. STATA version 16.0 (StataCorp LP, College Station, TX, USA) was used for statistical analyses. Statistical significance was defined as a two-sided *p* value < 0.05.

## 3. Results

The mean (SD) age of study participants at baseline was 39.7 (10.6) years, and 52.8% were male (Table 1). The prevalence of mild HL and moderate-to-severe HL were 2.8% and 0.6%, respectively. HL categories were positively associated with age, obesity, history of cancer, history of cardiovascular disease, hypertension, diabetes, medication for dyslipidemia, worse lipid profiles, hsCRP, and HOMA-IR. However, HL categories were inversely associated with education level and current smoking status (Table 1).

**Figure 2:**
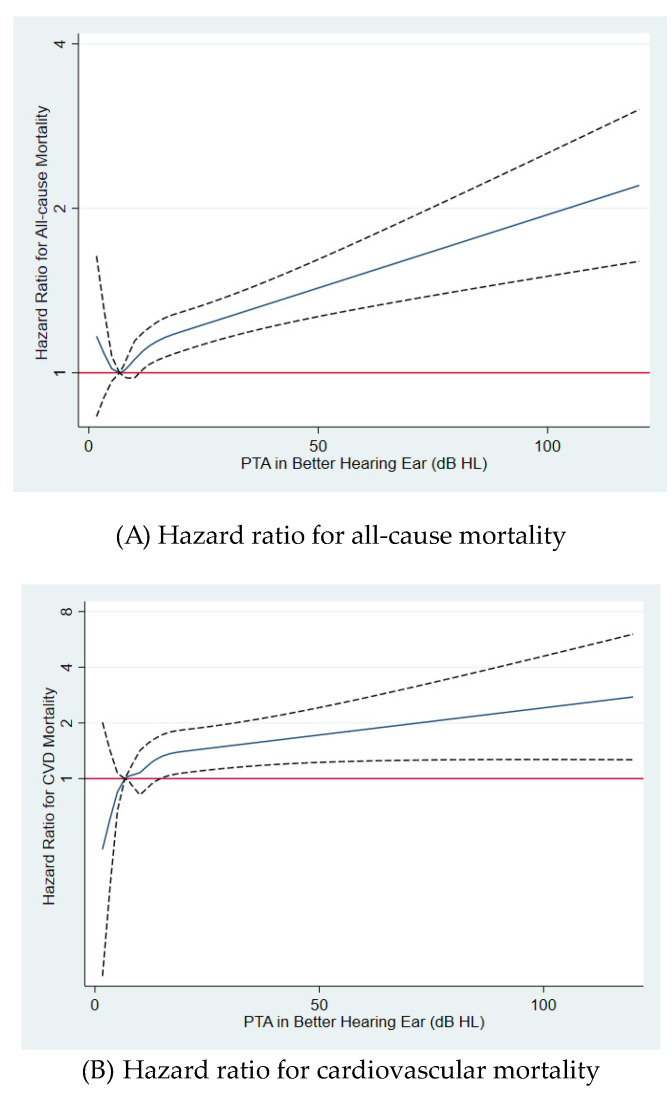

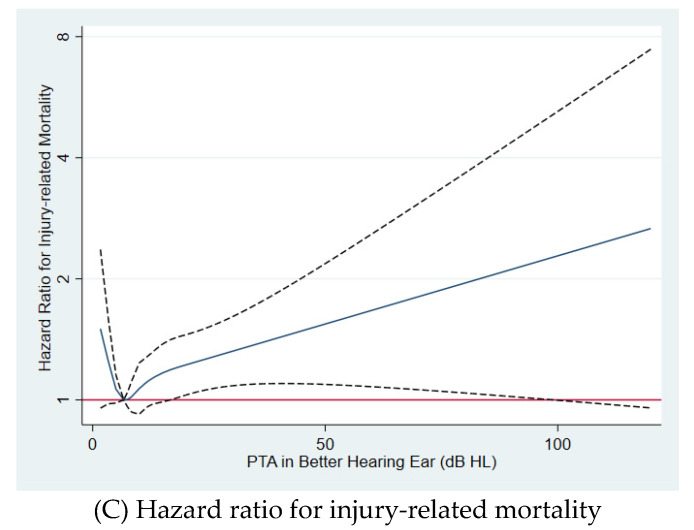
Multivariable-adjusted hazard ratios for (**A**) all-cause, (**B**) cardiovascular disease, and (**C**) injury-related mortality by hearing level. Estimates were adjusted by age (timescale), sex, center, year of screening exam, smoking status, alcohol intake, regular exercise, BMI, education level, exposure to occupational noise, history of diabetes, history of hypertension, history of cancer, history of cardiovascular disease, and medication for dyslipidemia. The solid line represents relative hazard, and the dashed lines represent the confidence intervals for the spline model. The horizontal red line corresponds to the normal reference hazard ratio of 1.0. dB, decibels; HL, hearing level; PTA, pure-tone average.

During the median follow-up of 8.4 years (up to 17 years; mean, 9.1 years; interquartile range, 5.3–13.2 years), 6581 overall deaths, 977 cardiovascular deaths, and 1161 injury-related deaths occurred (mortality rate of 124.8, 18.5, and 22.0 per 10^5^ person-years, respectively). HL was positively associated with overall, cardiovascular, and injury-related mortality. After adjustment for sex, study center, year of exam, smoking status, alcohol consumption, regular exercise, BMI, education attainment, history of diabetes, history of hypertension, history of cancer, history of cardiovascular disease, and medication for dyslipidemia, the multivariable-adjusted HRs (95% CIs) for overall mortality comparing participants with mild and moderate-to-severe HL to those with normal hearing were 1.13 (1.05–1.21) and 1.30 (1.16–1.46), respectively (Table 2, multivariable-adjusted model). Corresponding HRs (95% CIs) for cardiovascular mortality were 1.32 (1.10–1.58) and 1.53 (1.16–2.01), respectively, and corresponding HRs (95% CIs) for injury-related mortality were 1.03 (0.81–1.31) and 1.64 (1.13–2.36), respectively. When changes in HL and confounders during follow-up were updated as time-varying covariates, similar associations of HL with overall, cardiovascular, and injury-related mortality were observed. In spline regression analyses, there was a significant dose–response relationship of hearing level with overall, cardiovascular, and injury-related mortality (Figure 2).

In sensitivity analyses using HL based on either the left or right ear instead of the better ear, similar associations were observed (Table S1). In subgroup analyses, the association between HL and mortality did not differ by exposure to occupational noise (no vs. yes) (Table S2). The associations between HL and overall, cardiovascular, and injury-related mortality were consistently observed in participants free of both cancer and other chronic conditions such as diabetes and hypertension.

## 4. Discussion

In this large-scale cohort study, individuals with HL were at an increased risk for overall, injury-related, and cardiovascular mortality. The risk of all-cause and cardiovascular mortality increased with severity of HL, whereas the risk of injury-related mortality was significantly higher only in moderate-to-severe HL. These associations remained statistically significant even after adjusting for a wide range of confounders, including occupational noise exposure, and cardiovascular risk factors. Our findings indicate that individuals with HL may be at increased risk cardiovascular and injury-related deaths. 

Previous epidemiologic studies have examined the relationship of HL with overall mortality, but the findings are inconsistent [7,8,9,10,11,12,13,14,15]. The discrepant findings across studies may be attributable to differences in sample size, study population characteristics (e.g., age, sex, and ethnicity), methods of hearing status (self-report vs. audiometric tests), categorization or definition of HL, use of reference group, and adjustment for comorbidities and other confounders. Additionally, there are limited research on the association between HL and cause-specific mortality including cardiovascular mortality. A study of 4926 adults 67 years or older in Iceland reported an association of HL, defined HL as at least 35 dB hearing level for the pure-tone average (0.5–4 kHz) in the better ear, with significantly increased cardiovascular mortality and with a non-significant elevated risk of overall mortality [33]. Another recent study of 50,462 Norwegians with an average age of 51 years demonstrated a positive association of HL, defined HL as pure-tone average (0.5–4 kHz) ≥25 dB in the better ear, with significantly increased risk of overall and cardiovascular mortality, but not with injury-related mortality [34]. The present study included 580,798 participants of a relatively young population (average age: 39.7) with median follow-up of 8.1 years who underwent repeated audiometric tests and other screening examinations. A significantly increased risk of all-cause and cardiovascular mortality was observed in participants with HL, with graded association starting from mild HL, whereas the risk of injury-related mortality was significantly elevated with moderate-to-severe HL. The positive and significant relationship of HL with overall, injury-related, and cardiovascular mortality persisted even after adjustment for multiple confounders, including cardiovascular risk factors. 

Most prior studies did not incorporate these changes over time, even though hearing status and cardiovascular risk factors can change over time during follow-up. In contrast, the associations of HL with overall, injury-related, and cardiovascular mortality were consistently observed in our study, even when newly developed HL and changes in covariates during follow-up were considered as time-varying covariates. 

The exact mechanism of the observed association between HL and increased mortality is not well understood yet, but several explanations are possible. HL has been associated with difficulties in postural control, social isolation, depression, cognitive dysfunction, falls, and injuries [3,4,5,6,35]. People with more than moderate HL are more likely to be socially isolated and may have a poorer understanding of their own health issues and treatments [36]. All of these factors may contribute to increased overall and injury-related mortality. Additionally, epidemiologic studies have shown that established cardiovascular disease and cardiovascular risk factors such as hypertension, cardiovascular disease, smoking, hyperlipidemia, and diabetes mellitus are associated with HL [37,38]. The internal auditory artery (IAA), which supplies the cochlea and has no collateral anastomotic network, is highly susceptible to ischemia [39]. Histologic arteriosclerotic changes were found throughout the auditory system in older people, and microvascular disease involving the stria vascularis in the lateral wall of the cochlea can coexist with systemic vascular disease, which closely relates to cardiovascular risk factors [40,41]. Interestingly, subclinical atherosclerosis measured in the carotid intima was associated with 5-year incidence of hearing impairment in a predominantly middle-aged cohort [41]. In another study, the configuration of the audiometric pattern in patients with vascular disease, artery disease, myocardial infarction, strokes, and transient ischemic attack is typically low-frequency HL [21]. These pieces of evidence suggest that HL may reflect cardiovascular health and be affected by cardiovascular risk factors as well as subclinical and clinical cardiovascular diseases [41]. These factors explain the association between HL at low frequency range and cardiovascular mortality in our study. Further studies are required to confirm the relationship of hearing loss with incident cardiovascular disease and to understand the mechanisms underlying the higher risk of overall and cardiovascular mortality in patients with HL.

This study had some strengths, in terms of its large sample size, cohort design, detailed and standardized clinical and laboratory data at both the baseline and follow-up visits, availability of cause-specific mortality, and objective audiometric measures for hearing. This provided less bias compared to self-report hearing impairment methods used in previous studies. Our findings were also from a relatively healthy young and middle-aged population (average age: 39.7 years) with a lower rate of HL (3.4%). Therefore, the findings may have not been affected by survivor bias and biases related to comorbidities and medication use than previous research conducted among elderly populations.

## 5. Limitations

This study had several limitations. First, high-frequency HL above 2.0 kHz frequency was unable to be measured. In our study, HL was determined on the basis of pure tone audiometry across the usual speech spectrum (0.5, 1.0, and 2.0 kHz), but this range is important for conversational speech sounds and might better reflect the impact of hearing impairment on health outcomes in real life. Furthermore, the configuration of the audiometric pattern was typically affected at low-frequency HL in patients with vascular diseases [21]. This might explain why, in our study, HL at low frequency range showed a stronger association with cardiovascular mortality than death due to other causes. Second, information on noise exposure, a major cause of HL, was only available regarding workplace exposure but not leisure-time exposure. Excessive noise is mainly associated with high frequency hearing impairment above 3.0 kHz HL. Third, information on ototoxic drugs was not available. When subjects with history of cancer, diabetes, and hypertension were excluded, the association between HL and mortality was consistently observed in participants free of either cancer or other chronic conditions such as diabetes and hypertension. Fourth, information on lifestyle factors including physical activity and medical history was obtained via a self-administered structured questionnaire used in health checkup programs in Korea as a part of the National Health Insurance plan [27]. Thus, the possibility of residual confounding related to measurement errors and unmeasured confounders in observed association of the present study cannot be excluded. Lastly, our study subjects were young and middle-aged Korean adults attending regular health-checkup program, and the findings cannot be generalizable to other populations with different characteristics, such as age and race/ethnicity.

## 6. Conclusions

We demonstrated that HL was associated with an increased risk of overall, injury-related, and cardiovascular mortality in this cohort study of relatively young adults. Notably, even mild HL was significantly associated with moderately increased risk of cardiovascular mortality, even after taking into account confounders such as cardiovascular risk factors. Our findings support that mild HL, which can easily go unrecognized, may reflect cardiovascular health and may be an independent predictor of cardiovascular mortality. Further studies are required to determine whether proper screening, preventive measures, and treatments for cardiovascular diseases in patients with HL are needed to reduce cardiovascular mortality.

## Figures and Tables

**Table 1:** Baseline characteristics according to hearing loss category.

Characteristics	Overall	Hearing Loss Category			*p*-Value for Trend
		<25 dB	25 to <40 dB	≥ 40 dB	
Number	580,798	560,913	16,521	3364	
Age (years) ^a^	39.7 (10.6)	39.1 (10.0)	56.9 (11.8)	61.3 (12.6)	<0.001
Male (%)	52.8	52.8	51.5	53.1	0.020
Current smoker (%)	23.7	23.7	21.4	19.6	<0.001
Alcohol intake (%)	18.5	18.5	19.8	18.5	0.002
Regular exercise (%) ^b^	15.0	14.8	20.0	20.0	<0.001
High education level (%) ^c^	72.8	74.0	36.3	26.7	<0.001
History of cancer	1.5	1.6	3.3	4.5	<0.001
History of CVD	3.4	3.1	9.2	10.9	<0.001
Hypertension (%)	14.9	14.0	39.9	45.1	<0.001
Diabetes (%)	4.0	3.6	14.2	17.8	<0.001
Medication for dyslipidemia (%)	2.1	1.9	7.3	8.0	<0.001
Obesity (%) ^d^	28.8	28.4	39.3	36.2	<0.001
Body mass index (kg/m^2^)	23.3 (3.3)	23.3 (3.3)	24.3 (3.1)	24.1 (3.1)	<0.001
Systolic BP (mmHg) ^a^	112.2 (14.2)	111.9 (14.1)	120.2 (16.2)	121.9 (16.8)	<0.001
Diastolic BP (mmHg) ^a^	72.0 (10.2)	71.9 (10.2)	75.9 (10.4)	75.8 (10.3)	<0.001
Glucose (mg/dl) ^a^	94.8 (16.3)	94.5 (15.8)	102.5 (24.9)	104.4 (26.5)	<0.001
Total cholesterol (mg/dl) ^a^	193.8 (35.0)	193.6 (34.8)	202.0 (37.7)	200.2 (39.7)	<0.001
LDL-C (mg/dl) ^a^	116.0 (31.4)	115.8 (31.3)	122.2 (32.9)	121.8 (33.5)	<0.001
HDL-C (mg/dl) ^a^	57.4 (14.3)	57.5 (14.3)	54.4 (13.3)	54.0 (13.4)	<0.001
Triglycerides (mg/dl) ^e^	95 (67–142)	94 (66–141)	116 (82–168)	116 (83–166)	<0.001
hsCRP (mg/L) ^e^	0.4 (0.2–1.0)	0.4 (0.2–1.0)	0.6 (0.3–1.4)	0.7 (0.3–1.6)	<0.001
HOMA-IR ^e^	1.54 (1.03–2.17)	1.53 (1.02–2.16)	1.75 (1.18–2.46)	1.74 (1.13–2.51)	<0.001

Data are expressed as ^a^ mean (standard deviation), or percentage; ^b^ ≥ 3 times/week; ^c^ ≥ college graduate; ^d^ body mass index (BMI) ≥25 kg/m^2^; ^e^ median (interquartile range).

**Table 2:** Hazard ratios (95% CIs) for all-cause, cardiovascular, and injury-related mortality by pure-tone average of thresholds at 0.5, 1.0, and 2. 0 kHz in the better ear.

Hearing Category	Person-Years (PY)	Number of Events	Mortality Rate (10^5^ PY)	Age and Sex-Adjusted HR (95% CI)	Multivariable-Adjusted HR ^a^ (95% CI)	HR (95% CI) ^b^ in Model Using Time-Dependent Variables
All-cause mortality						
< 25 dB	5,085,564.9	5277	103.8	1.00 (reference)	1.00 (reference)	1.00 (reference)
25 to <40 dB	158,784.4	962	505.9	1.18 (1.09–1.27)	1.13 (1.05–1.21)	1.22 (1.13–1.31)
≥ 40 dB	29,201.7	342	1171.2	1.37 (1.22–1.54)	1.30 (1.16–1.46)	1.40 (1.25–1.57)
*p* for trend				<0.001	<0.001	0.001
Cardiovascular mortality						
< 25 dB	5,085,564.9	733	14.4	1.00 (reference)	1.00 (reference)	1.00 (reference)
25 to <40 dB	158,784.4	179	112.7	1.42 (1.18-1.69)	1.32 (1.10–1.58)	1.40 (1.18–1.67)
≥ 40 dB	29,201.7	65	222.6	1.59 (1.21–2.08)	1.53 (1.16–2.01)	1.78 (1.37–2.30)
*p* for trend				<0.001	<0.001	0.001
Injury-related mortality						
< 25 dB	5,085,564.9	1045	20.5	1.00 (reference)	1.00 (reference)	1.00 (reference)
25 to <40 dB	158,784.4	83	52.3	1.09 (0.86–1.39)	1.03 (0.81–1.31)	1.17 (0.93–1.47)
≥ 40 dB	29,201.7	33	113.0	1.80 (1.24–2.59)	1.64 (1.13–2.36)	1.73 (1.21–2.47)
*p* for trend				0.012	0.047	0.003

^a^ Hazard ratios (HRs) and 95% confidence intervals (CIs) were estimated from Cox proportional hazard models using age as timescale. Multivariable model was adjusted for age (timescale), sex, center, year of screening exam, smoking status, alcohol intake, regular exercise, BMI, education level, exposure to occupational noise, history of diabetes, history of hypertension, history of cancer, history of cardiovascular disease, and medication for dyslipidemia. ^b^ Estimated from Cox proportional hazard models with hearing threshold category, alcohol consumption, smoking status, regular exercise, BMI, history of diabetes, history of hypertension, history of cancer, history of cardiovascular disease, and medication for dyslipidemia as time-dependent categorical variables, and baseline age, sex, center, year of screening exam, education level, and exposure to occupational noise as time-fixed variables. BMI, body mass index; CI, confidence interval; HR, hazard ratio.

## Supplementary Materials

The following are available online at https://www.mdpi.com/2077-0383/9/5/1415/s1, Table S1: Hazard ratios (95% CIs) for all-cause, cardiovascular, and injury-related mortality by pure-tone average of thresholds at 0.5, 1.0, and 2. 0 kHz in either right or left ear, Table S2: Hazard ratios (95% CIs) for all-cause, cardiovascular, and injury-related mortality by hearing loss category and exposure to occupational noise.

## Abbreviations

ALT	alanine aminotransferase
AST	aspartate aminotransferase
BMI	body mass index
CI	confidence interval
HL	hearing loss
HOMA-IR	homeostasis model assessment of insulin resistance
HR	hazard ratio
HDL-C	high-density lipoprotein cholesterol
hsCRP	high-sensitivity C-reactive protein
LDL-C	low-density lipoprotein cholesterol
BP	blood pressure
hsCRP	high-sensitivity C-reactive protein
