# Supplementary material for: Hearing Loss and Risk of Overall, Injury-Related, and Cardiovascular Mortality: The Kangbuk Samsung Health Study
Source: J Clin Med. 2020 May 10;9(5):1415. doi: 10.3390/jcm9051415 (PMC7290521; doi:10.3390/jcm9051415)
Supplement: Supplementary file 1 [file jcm-09-01415-s001.pdf]

Table S1. Hazard ratios (95% CIs) for all-cause, cardiovascular, and injury-related mortality by pure-tone average of thresholds at 0.5, 1.0, and 2. 0 kHz in either right or left ear.

Hearing category	Person-years (PY)	Number of events	Mortality rate (10^5 PY)	Age and sex-adjusted HR (95% CI)	Multivariable-adjusted HR[a] (95% CI)	HR (95% CI)[b] in model using time-dependent variables
**All-cause mortality**						
**< 25 dB**	4,809,413.2	4,389	91.3	1.00 (reference)	1.00 (reference)	1.00 (reference)
**25 to <40 dB**	332,193.3	1,334	401.6	1.20 (1.12-1.28)	1.15 (1.08-1.23)	1.18 (1.11-1.27)
**≥ 40 dB**	131,944.5	858	650.3	1.25 (1.24-1.46)	1.28 (1.18-1.38)	1.35 (1.24-1.46)
**P for trend**				<0.001	<0.001	<0.001
**Cardiovascular mortality**						
**< 25 dB**	4,809,413.2	586	12.2	1.00 (reference)	1.00 (reference)	1.00 (reference)
**25 to <40 dB**	332,193.3	233	70.1	1.46 (1.24-1.72)	1.36 (1.15-1.60)	1.33 (1.13-1.57)
**≥ 40 dB**	131,944.5	158	119.7	1.64 (1.35-2.00)	1.55 (1.27-1.89)	1.62 (1.34-1.96)
**P for trend**				<0.001	<0.001	<0.001
**Injury-related mortality**						
**< 25 dB**	4,809,413.2	933	19.4	1.00 (reference)	1.00 (reference)	1.00 (reference)
**25 to <40 dB**	332,193.3	146	44.0	1.18 (0.98-1.42)	1.12 (0.93-1.35)	1.17 (0.98-1.41)
**≥ 40 dB**	131,944.5	82	62.1	1.39 (1.09-1.77)	1.29 (1.01-1.64)	1.37 (1.08-1.73)
**P for trend**				0.003	0.033	0.005

[a] Estimated from Cox proportional hazard models using age as timescale were used to estimate hazard ratios (HRs) and 95% confidence intervals (CIs). Multivariable model was adjusted for age (timescale), sex, center, year of screening exam, smoking status, alcohol intake, regular exercise, BMI, education level, exposure to occupational noise, history of diabetes, history of hypertension, history of cancer, history of cardiovascular disease, and medication for dyslipidemia.

[b] Estimated from Cox proportional hazard models with hearing threshold category, alcohol consumption, smoking status, regular exercise, BMI, history of diabetes, history of hypertension, history of cancer, history of cardiovascular disease, and medication for dyslipidemia as time-dependent categorical variables and baseline age, sex, center, year of screening exam, education level, and exposure to occupational noise as time-fixed variables.

BMI, body mass index; CI, confidence interval; HR, hazard ratio.

Table S2. Hazard ratios (95% CIs) for all-cause, cardiovascular, and injury-related mortality by hearing loss category and exposure to occupational noise.

**Hearing category**	**Multivariable-adjusted HR[a] (95% CI)**		**P for interaction**
	No exposure to occupational noise (N=522,234)	Exposure to occupational noise (N=58,564)	
**All-cause mortality**			0.49
**< 25 dB**	1.00 (reference)	1.00 (reference)	
**25 to <40 dB**	1.11 (1.03-1.20)	1.41 (0.96-2.07)	
**≥ 40 dB**	1.29 (1.14-1.45)	1.39 (0.75-2.56)	
**P for trend**	<0.001	0.069	
**Cardiovascular mortality**			0.10
**< 25 dB**	1.00 (reference)	1.00 (reference)	
**25 to <40 dB**	1.27 (1.06-1.53)	2.99 (1.38-6.51)	
**≥ 40 dB**	1.50 (1.14-1.98)	1.91 (0.45-8.17)	
**P for trend**	<0.001	0.024	
**Injury-related mortality**			0.55
**< 25 dB**	1.00 (reference)	1.00 (reference)	
**25 to <40 dB**	0.99 (0.78-1.27)	1.77 (0.64-4.93)	
**≥ 40 dB**	1.60 (1.10-2.33)	1.47 (0.20-10.68)	
**P for trend**	0.091	0.311	

[a] Estimated from Cox proportional hazard models using age as timescale were used to estimate hazard ratios (HRs) and 95% confidence intervals (CIs). Multivariable model was adjusted for sex, center, year of screening exam, smoking status, alcohol intake, regular exercise, BMI, education level, history of diabetes, history of hypertension, history of cancer, history of cardiovascular disease, and medication for dyslipidemia.
